# High-Speed Electrospinning of Ethyl Cellulose Nanofibers via Taylor Cone Optimization

**DOI:** 10.1021/acsaenm.4c00527

**Published:** 2024-10-02

**Authors:** Qiangjun Hao, John Schossig, Adedayo Towolawi, Kai Xu, Erwan Bayiha, Mayooran Mohanakanthan, Derek Savastano, Dhanya Jayaraman, Cheng Zhang, Ping Lu

**Affiliations:** † Department of Chemistry and Biochemistry, Rowan University, Glassboro, New Jersey 08028, United States; ‡ Chemistry Department, Long Island University (Post), Brookville, New York 11548, United States

**Keywords:** ethyl cellulose, high-speed electrospinning, Taylor cone optimization, sheath liquid assistance, porous nanofibers

## Abstract

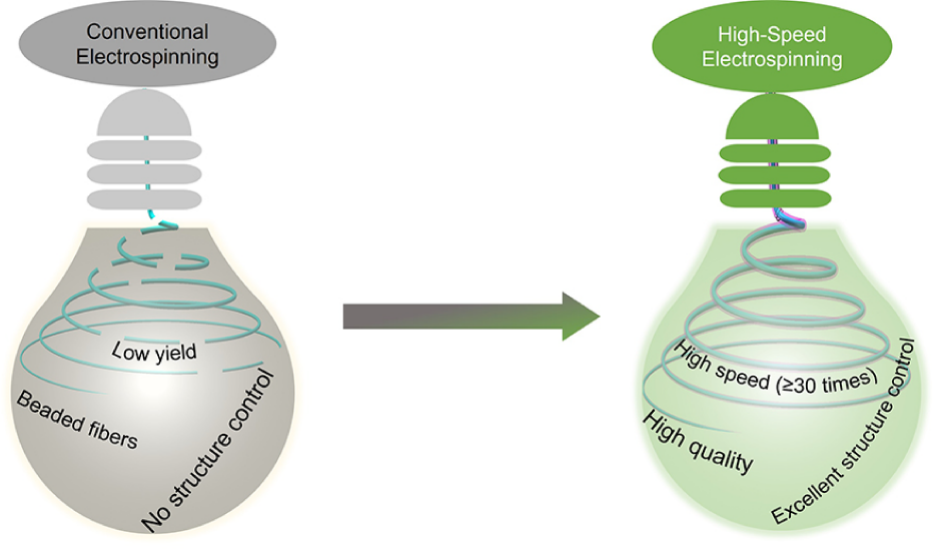

Ethyl cellulose (EC) is one of the most widely used cellulose derivatives. Nevertheless, challenges such as the formation of beaded fibers, low yield, and nonporous internal structure persist in electrospinning, limiting functional improvements and industrial applications. This study invented a groundbreaking high-speed electrospinning technique through sheath liquid assistance to optimize the Taylor cone, dramatically enhancing the yield, morphology, and formation of porous structures of EC nanofibers beyond what has been seen in the literature to date. Our study emphasizes the crucial role of the sheath liquid’s physical and chemical properties in controlling the morphology and diameter of EC nanofibers. It was discovered that highly polar and viscous sheath liquids led to the formation of beaded structures. Most importantly, the sheath liquid-assisted method substantially increased the ejection rate of the EC solution tens and hundreds of times compared to the current low-speed electrospinning method (0.1–1 mL/h) by refining the shape of the Taylor cone and resolving low productivity challenges in conventional nanofiber production. Meanwhile, increasing the flow rate of the EC or the sheath liquid accelerated the phase separation of EC solutions, thereby promoting the formation of porous structures in EC nanofibers. A pronounced porous structure was observed when the core EC flow rate reached 25 mL/h or the sheath chloroform flow rate reached 20 mL/h. Furthermore, our sheath liquid-assisted high-speed electrospinning technique demonstrated universal applicability to ECs with varying molecular weights. This study comprehensively addressed challenges in controlling the yield, morphology, and internal structure of EC nanofibers through sheath-solution-assisted high-speed electrospinning technology. These findings provide an innovative approach to developing next-generation electrospinning technologies to enhance the yield and properties of natural polymers for sustainability.

## Introduction

Ethyl cellulose (EC) is a linear polysaccharide derived from cellulose, consisting of cellulose backbones with partial replacement of hydrogen in the cellulose hydroxyl end groups by ethyl end groups.^1−4^ The excellent mechanical properties, low cost, and renewability of EC make it one of the most widely used cellulose derivatives.^5,6^ EC is biocompatible and approved by the United States Food and Drug Administration as a generally-recognized-as-safe chemical substance in biomedical applications.^7^ Due to good biocompatibility, EC nanofibers have been widely used as carriers for drug transport.^8−10^ In environmental applications, EC nanofibers that graft fluorescent molecules can preserve environmental pollutants’ detection ability while leveraging EC’s excellent biocompatibility and biodegradability.^5,11^ Additionally, EC is used as a binder in printing pastes, electrets, and thermoelectric materials.^12^ Electrospinning is an electrohydrodynamic atomization method widely acknowledged for its simplicity and versatility in generating continuous nanofibers and creating 3D constructs with hierarchical porosity through organized or random stacking of nanofibers.^13−16^ Previous works have addressed the electrospinnability of EC solutions.^17^ Generally, neat EC solutions exhibited high conductivity, high surface tension, and high viscosity. These properties resulted in the formation of irregular particulates interwoven with nonuniform nanofibers.^18^ Therefore, controlling the morphology and diameter of EC nanofibers in electrospinning presents challenges, as the formation of morphological defects such as beaded fibers, beads, and inconsistent fiber sizes frequently occurs.^19^ Besides, EC fibers with micro- and nanoporous structures, highly desired in various applications, have not been reported. According to some reports, EC nanofibers fabricated via the electrospinning process mostly exhibit a smooth surface morphology.^20,21^ Finally, the most critical challenge of the conventional electrospinning process is its low production rate. The average production rate of a lab-scale electrospinning process is around 0.1–0.2 g/h from a single spinneret, which dramatically limits the application of EC nanofibers.^22−24^

Numerous methods have been utilized to address these limitations and to enhance the properties of EC nanofibers. A second polymer has been commonly added as a processing aid to facilitate the formation of uniformly thin EC nanofibers.^25−27^ Lim et al. evaluated poly(ethylene oxide) (PEO) as a processing aid to enhance the electrospinnability of EC solutions. The fibers were fabricated using the needleless free-liquid-surface electrospinning method, which is more conducive to scale-up production than the typical spinneret approach.^18^ Yang et al. reported the fabrication of EC/poly(vinylpyrrolidone) (PVP) fibers with porous structures throughout using centrifugal spinning with a binary solvent system of ethanol and water.^28^ Optimizing the solvent is also a common strategy for improving the properties of EC fibers. Huang et al. investigated the effects of a multicomponent solvent system on the diameter distribution and surface morphology of EC fibers. The results demonstrate that regular holes were formed on the surface of fibers from pure tetrahydrofuran (THF) and an 80% THF solution in dimethylacetamide (DMAc), while a smooth surface was observed for pure DMAc and an 80:20 DMAc–THF ratio. However, only a few pores were observed on the surface.^29^ Unfortunately, no relevant work has been conducted to increase the EC nanofiber production rate from a single spinneret in electrospinning. Some recent works found the effect of a sheath liquid on the synthesis of EC fibers in the electrospinning process. Yu et al. found that the sheath liquid affected the size of EC fibers and that low-volatility liquid produced more beads.^30^ Huang et al. found that ethanol as a sheath liquid helped EC fibers encapsulate drugs. The presence of ethanol also prevented the EC solution from clogging during the electrospinning process.^31^ Although the methods mentioned above have contributed to the development of EC fibers to some extent, several challenges still have not been addressed, particularly the yield, uniformity, and structural control of EC nanofibers. Thus, the development of more efficient and accessible ways to quickly generate substantial amounts of consistent electrospun nanofibers with precisely tailored surface textures and internal structures remains a vital pursuit in nanofiber production.

In this study, we present a novel approach to improving the properties and significantly increasing the yield of EC nanofibers through a high-speed electrospinning process assisted by sheath liquids. Sheath liquids acted as a protective layer, shielding the polymer from direct exposure to air and thereby maintaining the stability and spinability of the EC solution even at an extremely high flow rate during electrospinning. By exploring various sheath liquids, we identified critical physical and chemical parameters of the sheath liquids—boiling point, polarity, and viscosity—that influence the stability of electrospinning and morphology of the resultant EC nanofibers. Nanofiber production yield has consistently been a significant challenge in the electrospinning of EC. Sheath liquid-assisted technology significantly increased the ejection rate of the EC solution through the Taylor cone optimization, leading to an increase in the production efficiency by tens to hundreds of times that of conventional methods. Remarkably, increasing the flow rate of the EC or the sheath liquid accelerated the phase separation of the EC liquid jet, enabling the formation of porous structures in EC nanofibers. This significantly enhances the functionality and the application potential of EC nanofibers. Furthermore, sheath liquid-assisted high-speed electrospinning worked for ECs with a wide range of molecular weights, demonstrating its broad applicability. These findings are essential for implementing sheath liquid-assisted high-speed technology in electrospinning, offering valuable experimental data for rapidly producing EC nanofibers of high quality and with well-controlled structures, which tackle the three primary hurdles in the fabrication of EC nanofibers: low production rate, formation of irregular structures, and absence of porosity.

## Experimental Section

### Chemicals and Materials

Polymeric excipient EC powders with different molecular weights (89 000, 130 000, 224 000, and 339 000 g/mol) and 48.0–49.5% (w/w) ethoxyl content were purchased from TCI America. The viscosities of 5% (w/v) solutions of these powders in 80:20 toluene/ethanol at 25 °C were determined to be 9–11, 18–22, 45–55, and 90–110 mPa·s, respectively. A variety of sheath liquids, including ethanol, methanol, acetone, tetrahydrofuran, chloroform, dichloromethane, and diethyl ether, were also purchased from TCI America. No further purification was conducted on the received chemicals. The water utilized in the experiments was purified using a Millipore Direct-Q 8 UV water purification system, achieving a resistivity of 18.2 MΩ·cm at 25 °C.

### Exploring the Influence of Sheath Liquids on the Morphology of EC Nanofibers

The coaxial electrospinning technique was used to examine the impact of different sheath liquids on the EC nanofiber morphology. A mixture of ethanol and water (8:2) was used as the solvent for the EC solution. In a typical experiment, 20% EC (89 000 g/mol, 9–11 mPa·s) was fed into the core needle of a metallic coaxial spinneret at 2 mL/h. Simultaneously, various liquids (i.e., water, ethanol, methanol, acetone, tetrahydrofuran, chloroform, dichloromethane, and diethyl ether) were delivered to the outer needle at 0.5 mL/h. Independent control over the flow rate of the core fluid (i.e., 20% EC solution) and outer fluid was achieved using two programmable syringe pumps (Legato 110, KD Scientific) operated through Adagio Syringe Pump Control Software (KD, Scientific). A high-voltage DC power supply (ES30P-5W, Gamma High Voltage Research) was connected to the stainless-steel coaxial spinneret. A 15 kV charge was applied to the spinneret, and a liquid jet comprising the core EC solution and sheath liquid was ejected from the Taylor cone. Subsequently, EC nanofibers were collected after the swift evaporation of the solvent and sheath liquid in a conductive collector positioned 20 cm below the needles’ tip. EC solution (20%) was also fed into the uniaxial spinneret at 2 mL/h as a blank control group. All electrospinning experiments were conducted at 25 ± 2 °C and 40 ± 3% relative humidity. The temperature was regulated by the laboratory’s central air conditioning system, and humidity was maintained by an industrial-grade humidifier/dehumidifier in the fume hood. Before further experiments and characterizations, the nanofibers obtained were dried in a vacuum oven at room temperature for 24 h.

### Investigating the Impact of Sheath Liquids on the Yield of EC Nanofibers

The coaxial electrospinning technique was employed to investigate the influence of different sheath liquids on the yield of EC nanofibers. Two volatile solvents, environmentally friendly ethanol and highly effective chloroform, were chosen as model sheath liquids to improve the yield of EC nanofibers. The 20% EC solution (9–11 mPa·s) was injected at different flow rates (1, 5, 10, 15, 20, 25, and 30 mL/h) into the core needle of a metallic coaxial spinneret. Simultaneously, ethanol or chloroform was injected into the outer needle at a rate of 0.5 mL/h. Other optimized parameters included a high voltage of 15 kV, a distance of 20 cm between the aluminum foil (EC nanofiber collector) and the coaxial spinneret nozzle, a temperature of 25 ± 2 °C, and a relative humidity of 40 ± 3%. The EC nanofibers obtained were subsequently dried at room temperature in a vacuum oven for 24 h. The weight of EC nanofibers was measured at various intervals (10, 20, 30, 40, 50, and 60 min) using a precision balance, and the final yield of EC nanofibers was documented with a camera. The change in the Taylor cone at different core EC and sheath liquid flow rates during electrospinning was monitored and recorded using a digital camera (EOS R1, Canon) equipped with a macro lens (EF 100 mm f/2.8L Macro IS USM, Canon) at approximately 100× magnification on a 27 in. computer monitor (27GL83A-B QHD IPS 1 ms with 144 Hz, LG).

### Examining the Broad Applicability of Sheath Liquid-Assisted High-Speed Electrospinning for High-Molecular-Weight ECs

EC powders of different molecular weights were formulated into EC solutions of different concentrations (20% for 18–22 and 45–55 mPa·s ECs, 8% for 90–110 mPa·s EC), and the solvent was a mixture of ethanol and water (8:2). EC solutions with different molecular weights/viscosities (89 000 g/mol/9–11 mPa·s, 130 000 g/mol/18–22 mPa·s, 224 000 g/mol/45–55 mPa·s, and 339 000 g/mol/90–110 mPa·s) were injected at 2 mL/h into the core needle of a metallic coaxial spinneret. Concurrently, chloroform was delivered as a sheath solution into the outer needle at a rate of 0.5 mL/h. The other electrospinning parameters were kept the same as those in the experiments described above. For comparison, the same procedures were repeated using the same core EC solution but without the sheath liquid. This resulted in no uniform nanofibers being obtained.

### Characterization

High-resolution field-emission scanning electron microscopy (SEM, Apreo S, FEI) was employed to examine EC nanofibers’ surface morphology and internal structure. To expose the cross sections of nanofibers, the synthesized EC nanofibers were first frozen in liquid nitrogen at −195.8 °C for 10–20 min. Afterward, a sharp blade was used to cut the frozen EC nanofibers to obtain cross-sectional views of the EC nanofibers. To further expose the internal structure, the epidermis of the EC nanofibers was partially removed using a mixture of ethanol and water in a separate experiment. No such pretreatments were used for general-purpose nanofiber imaging. All samples underwent sputter-coating with gold for 60 s to enhance their electrical conductivity. Representative SEM images of the samples were captured at a work distance of 6 mm, employing an accelerating voltage of 10 kV and a beam current of 0.40 nA. Nanofiber size measurements were performed by using ImageJ (NIH) based on the representative SEM images. The crystalline structures of the samples were analyzed via X-ray diffraction (XRD), employing a Bruker D8 Discover machine with Cu Kα radiation set at 40 kV and 40 mA. The scan parameters were set at 0.02° per step and 0.5 s per step at 2θ ranging from 5° to 90°. Infrared spectroscopy was carried out using the attenuated total reflection (ATR) method with a PerkinElmer Frontier spectrometer to determine the evaporation of solvent and sheath liquid as well as the effect of sheath liquid on the chemical composition of the resultant EC nanofibers. The absorbance spectra of the nanofibers were recorded in the wavenumber range of 4000 to 650 cm^–1^ with a spectral resolution of 4 cm^–1^. An average was taken from 128 scans for each sample. Furthermore, the mean diameters and standard deviations of the EC nanofibers were determined by analyzing over 100 individual nanofibers from representative SEM images. These measurements were processed and analyzed by using OriginPro software (OriginLab).

## Results and Discussion

### Improving EC Nanofiber Yield and Properties via Taylor Cone Optimization with Sheath Liquids Using Coaxial Electrospinning

**Figure 1:**
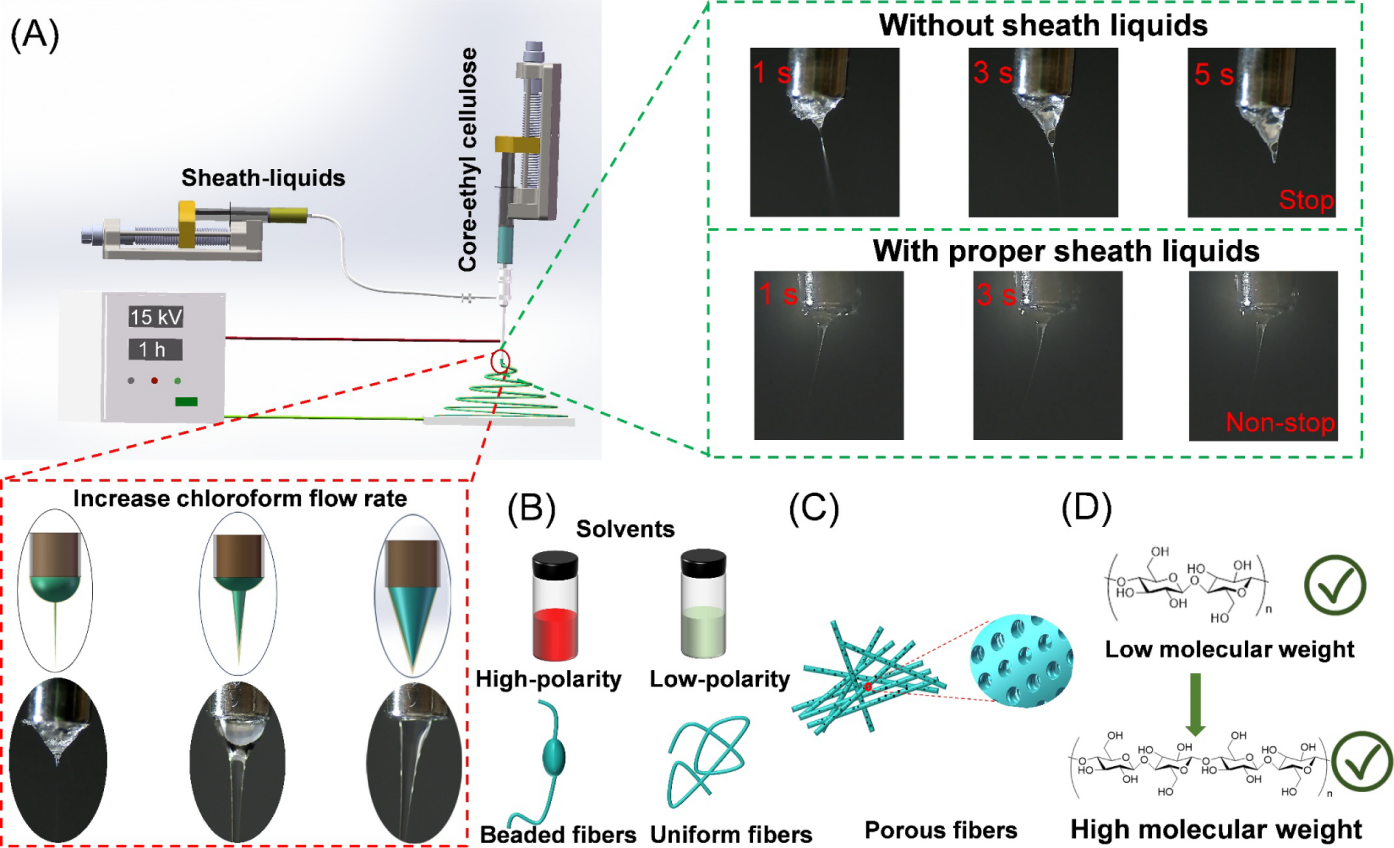
Schematic illustration showing how Taylor cone optimization improves the EC nanofiber yield and properties via high-speed electrospinning: (A) high-speed electrospinning process through sheath liquid assistance (right inset: comparison of the effect without and with sheath liquids; bottom inset: the Taylor cone alternations at increasing flow rates of sheath liquids), (B) effect of sheath liquid polarity on the morphology of EC nanofibers, (C) porous EC nanofibers generated from the high-speed electrospinning, and (D) wide-ranging effectiveness of the Taylor cone optimization technique to the electrospinning of ECs with different molecular weights.

Figure 1 presents a schematic illustration depicting the process of high-speed electrospinning through sheath liquid assistance to enhance the yield and properties of EC nanofibers. The EC nanofibers were fabricated by utilizing a coaxial electrospinning setup. In a standard process, different organic liquids were introduced into the outer needle of the metal coaxial spinneret as the sheath liquid (Figure 1A). The 20% EC solution was supplied to the inner needle. Independent control of the feed rates of the sheath liquids and EC solution was achieved using two programmable syringe pumps. For comparison, electrospinning of EC without sheath liquid was conducted, which stopped quickly due to the fast drying of the Taylor cone (Figure 1A, right inset). In the Taylor cone, strong hydrogen bonding among EC molecules accelerated the drying process.^32^ In contrast, the electrospinning of EC employing the sheath liquid continued for hours without any clogging because of the slower drying of the EC solution in the Taylor cone at the tip of the spinneret. Interestingly, we observed that the base of the Taylor cone (round part) contracted toward the needle, while the tip of the Taylor cone (sharp liquid ejection point) enlarged due to the the increasing flow rate of the sheath liquid, thus increasing the yield of the EC nanofibers exponentially (Figure 1A, bottom inset). We also discovered that the higher the polarity of the sheath liquid, the easier it penetrated the EC solution, thereby influencing the concentration of the EC solution during electrospinning. At low concentrations, the molecular chains in the EC solution are insufficiently entangled to form fibers, resulting in beaded fibers (Figure 1B).^33^ Furthermore, the increased flow rate of the EC solution accelerated phase separation during electrospinning, leading to the formation of EC nanofibers with a porous structure (Figure 1C). Importantly, the sheath liquid-assisted electrospinning technique successfully produced nanofibers from higher-molecular-weight EC solutions, which were previously not achievable with conventional electrospinning, highlighting its versatility and broad applicability (Figure 1D).

**Figure 2:**
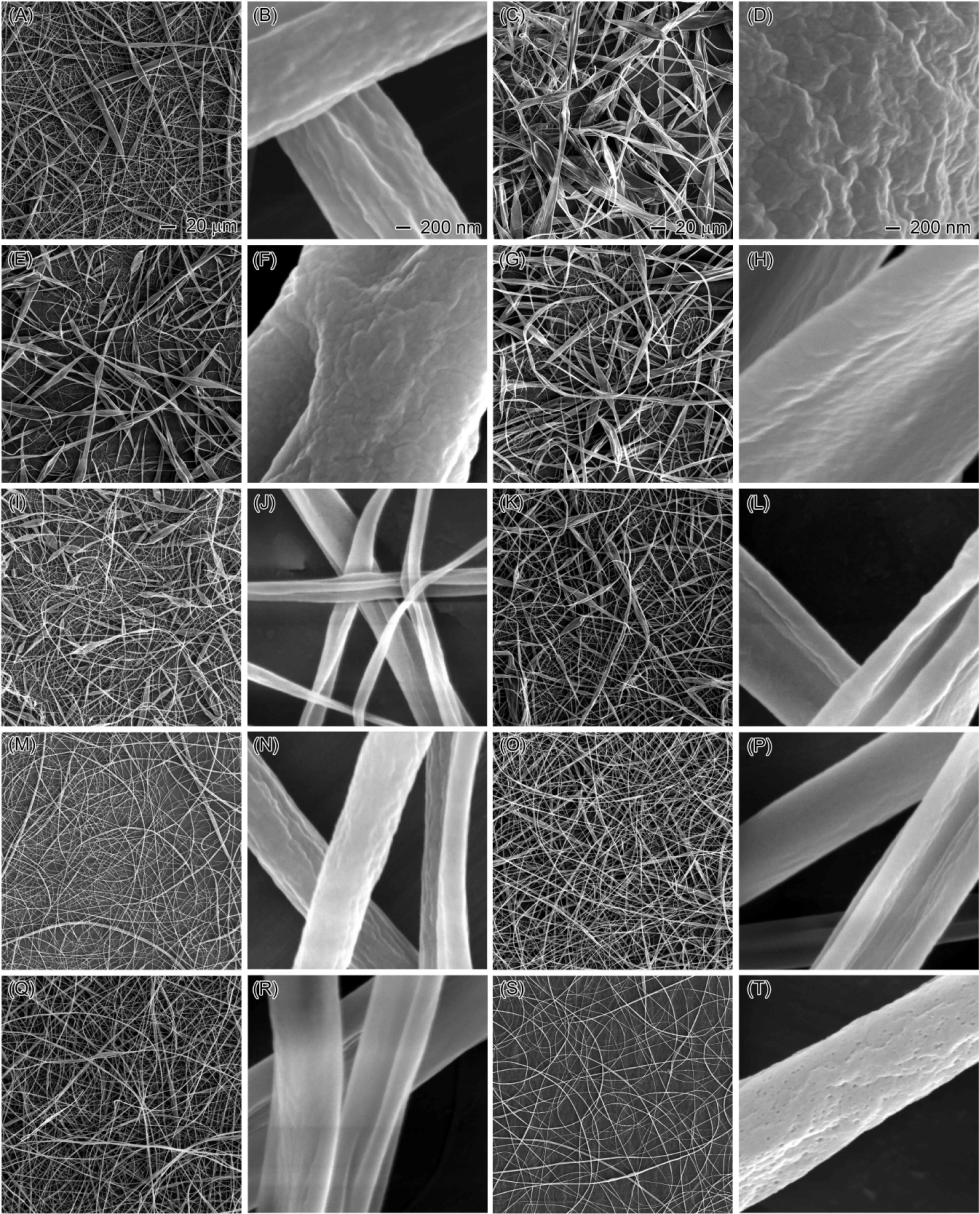
SEM images showing the effect of different sheath liquids on the EC nanofibers’ morphology: (A, B) no sheath liquid, (C, D) water, (E, F) cyclohexane, (G, H) methanol, (I, J) ethanol, (K, L) acetone, (M, N) tetrahydrofuran, (O, P) dichloromethane, (Q, R) chloroform, and (S, T) diethyl ether. The core fluid is a 20% EC solution in 2:8 (w:w) water and ethanol, and the sheath liquid is an anhydrous organic liquid (except pure water). The scale bars in panels A–D apply to the images in the same column.

The morphology of nanofibers directly affects their performance and applicability in various fields.^34−37^ The advantage of uniform fibers is their ability to provide consistent performance, enhanced processability, and versatility across applications, ultimately improving functionality, efficiency, and reliability in a variety of industrial and technical environments.^38,39^Figure 2 shows SEM images of EC nanofibers produced without sheath liquids or using different sheath liquids. Without sheath liquids, a small amount of beaded EC nanofibers with a smooth surface were obtained (Figure 2A,B). The formation of beaded fibers is usually related to the high surface tension and viscosity of the polymer solution.^13,40^ Additionally, without sheath liquids, electrospinning was frequently interrupted due to the fast drying of the EC solution in the Taylor cone. When solvents with high boiling points such as pure water (100 °C) and cyclohexane (81 °C) were used as the sheath liquid, solution dripping and clogging at the tip of the spinneret frequently occurred during the electrospinning process (Figure S1), the proportion of beaded nanofibers increased, and the surface became rougher (Figure 2C–F). The use of highly volatile and extremely polar sheath liquids (i.e., methanol, ethanol, and acetone) resulted in a smoother, clog-free electrospinning process. While the beaded structure of the EC nanofibers persisted, their surfaces became noticeably smoother (Figure 2G–L). To further investigate the influence of sheath liquid polarity on EC nanofibers, highly volatile and moderately polar solvents (i.e., tetrahydrofuran, dichloromethane, and chloroform) were employed as sheath liquids. This resulted in a smooth, uninterrupted electrospinning process, yielding uniform EC nanofibers (Figure 2M–R). Utilizing diethyl ether, an extremely volatile and nonpolar solvent, as the sheath liquid resulted in a smooth and unclogged electrospinning process. The EC nanofibers produced were of uniform size and have smooth surfaces with microporous structures (Figure 2S,T). The high volatility of diethyl ether promoted phase separation, contributing to the development of a porous surface structure in the EC nanofibers.

**Figure 3:**
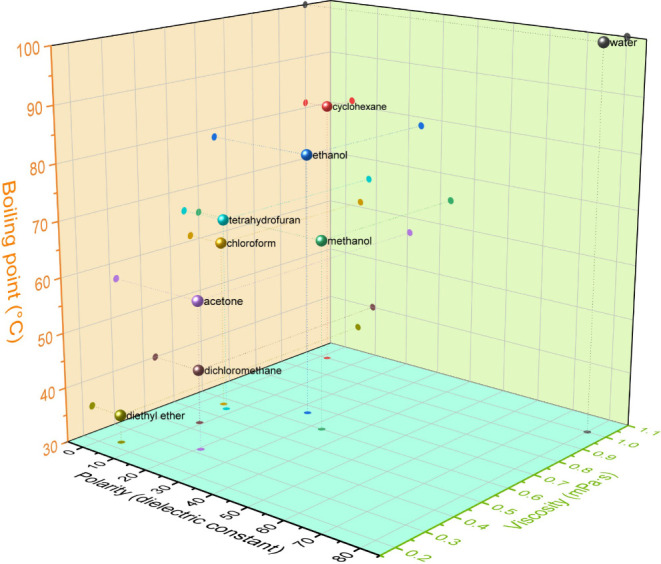
Physical and chemical properties of different organic solvents: polarity (dielectric constant), boiling point, and viscosity.

The results highlight the profound influence of sheath liquids on the morphologies of EC nanofibers. Due to their pivotal role in the electrospinning process, the physical and chemical properties of sheath fluids emerge as primary factors underlying the observed effect. Figure 3 illustrates the boiling points, dielectric constants (indicating polarity), and viscosities of various sheath liquids. Water and cyclohexane, with their high boiling points (or lower volatility), elevated viscosity and surface tension during electrospinning. This hindered solvent evaporation, leading to process instability, blockages, and dripping. Therefore, a high volatility (or low boiling point) is a crucial factor in selecting a suitable sheath liquid. Beyond volatility, polarity and viscosity also significantly influenced the EC nanofiber morphology. Sheath liquid polarity affected the core EC solution concentration due to rapid evaporation and diffusion, which is critical, as even minor concentration changes can drastically alter the nanofiber morphology. High EC concentrations increased viscosity, causing blockages, while low concentrations led to bead formation (only EC particles were observed at concentrations as low as 10%, Figure S2). The polarity of the sheath liquid influenced how easily it mixed with the EC solution, thereby affecting the EC concentration during electrospinning. Sheath liquids with higher polarity tended to readily mix with the EC solution, decreasing the concentration during electrospinning. Low concentrations resulted in insufficient entanglement of EC molecular chains, leading to bead formation instead of fibers.^41,42^ The viscosity of the sheath liquid also played a role by altering the surface tension of the EC solution. High-viscosity sheath liquids increased surface tension, hindering the continuous electrospinning process.^43^ The physical and chemical interactions between the sheath liquid and core EC solution were complex, with multiple factors contributing to the final nanofiber morphology rather than a single factor.

**Figure 4:**
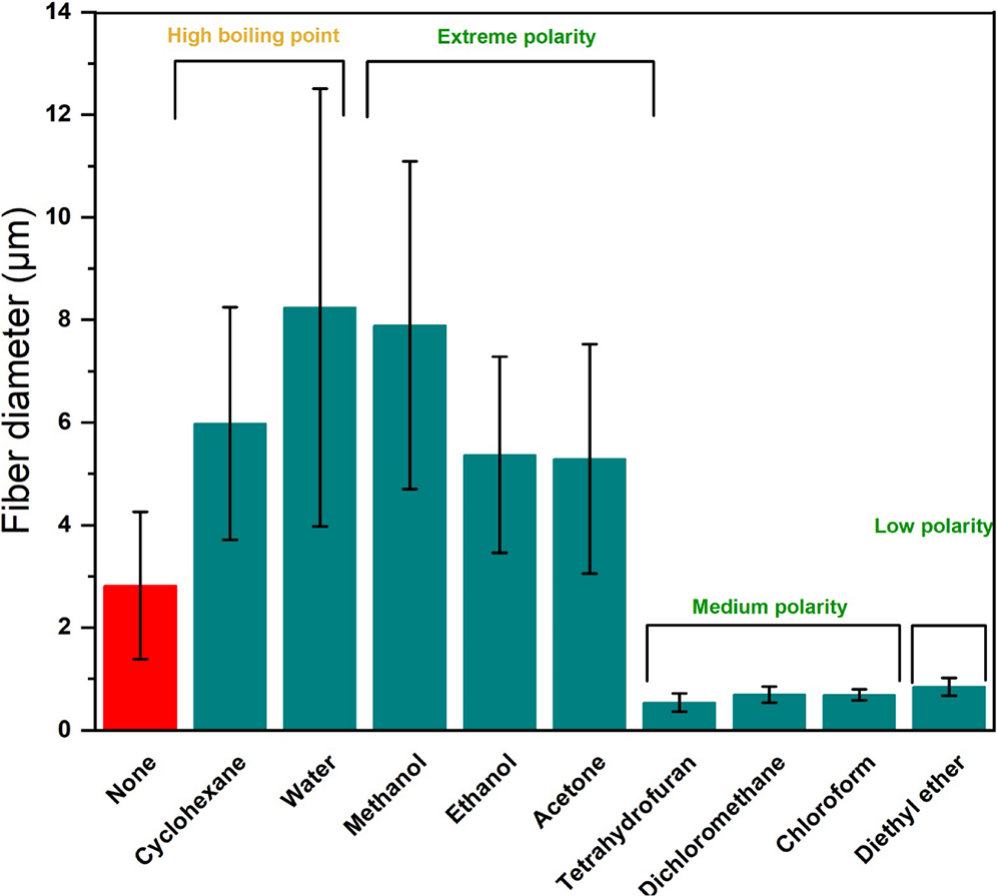
Diameter variations of EC nanofibers: impact of sheath liquids.

Precise control over the nanofiber diameter empowers the fine-tuning of critical properties such as surface area, mechanical strength, porosity, and permeability. This tailored customization enables the engineering of materials with specific performance characteristics, unlocking new possibilities across a wide range of applications.^44−46^Figure 4 demonstrates the effect of different sheath liquids on the diameter of EC nanofibers produced via coaxial electrospinning. Without a sheath liquid, the average diameter was 2.810 ± 1.430 μm. Using nonvolatile sheath liquids, EC nanofibers showed a significant increase in diameter. For example, the nanofiber diameters reached 8.243 ± 4.267 and 5.980 ± 2.265 μm when water and cyclohexane were used as the sheath liquid, respectively. Conversely, when highly volatile and extremely polar sheath liquids such as methanol, ethanol, and acetone were used, the diameters were 7.895 ± 3.193, 5.370 ± 1.914, and 5.291 ± 2.239 μm, respectively. When employing moderately polar and highly volatile sheath liquids like tetrahydrofuran, dichloromethane, and chloroform, the fiber diameters were significantly reduced to 0.539 ± 0.177, 0.695 ± 0.013, and 0.695 ± 0.158 μm, respectively. Utilizing diethyl ether—a highly volatile and nonpolar liquid—resulted in a fiber diameter of 0.846 ± 0.174 μm. The observed trend where nanofiber diameters increased with the increasing boiling point of the sheath liquid, which indicated its decreasing volatility, can be explained by several factors. First, low-volatile sheath liquids increased the viscosity and surface tension, which hindered the stretching of the polymer jet and resulted in thicker fibers.^47^ Second, these liquids slowed down solvent evaporation, delaying the curing and stretching of the polymer jet and producing coarser fibers.^48−50^ Third, as the polarity of the sheath liquid decreased, the uniformity of the fibers significantly improved, particularly noticeable using highly volatile sheath liquid, where the standard deviation of fiber diameters markedly decreased. These results highlight the effectiveness of sheath liquid-assisted electrospinning in precisely controlling fiber size and morphology and tailoring nanofiber properties to meet diverse application requirements.

### Developing High-Speed Electrospinning of EC with Minimal Sheath Liquids

**Figure 5:**
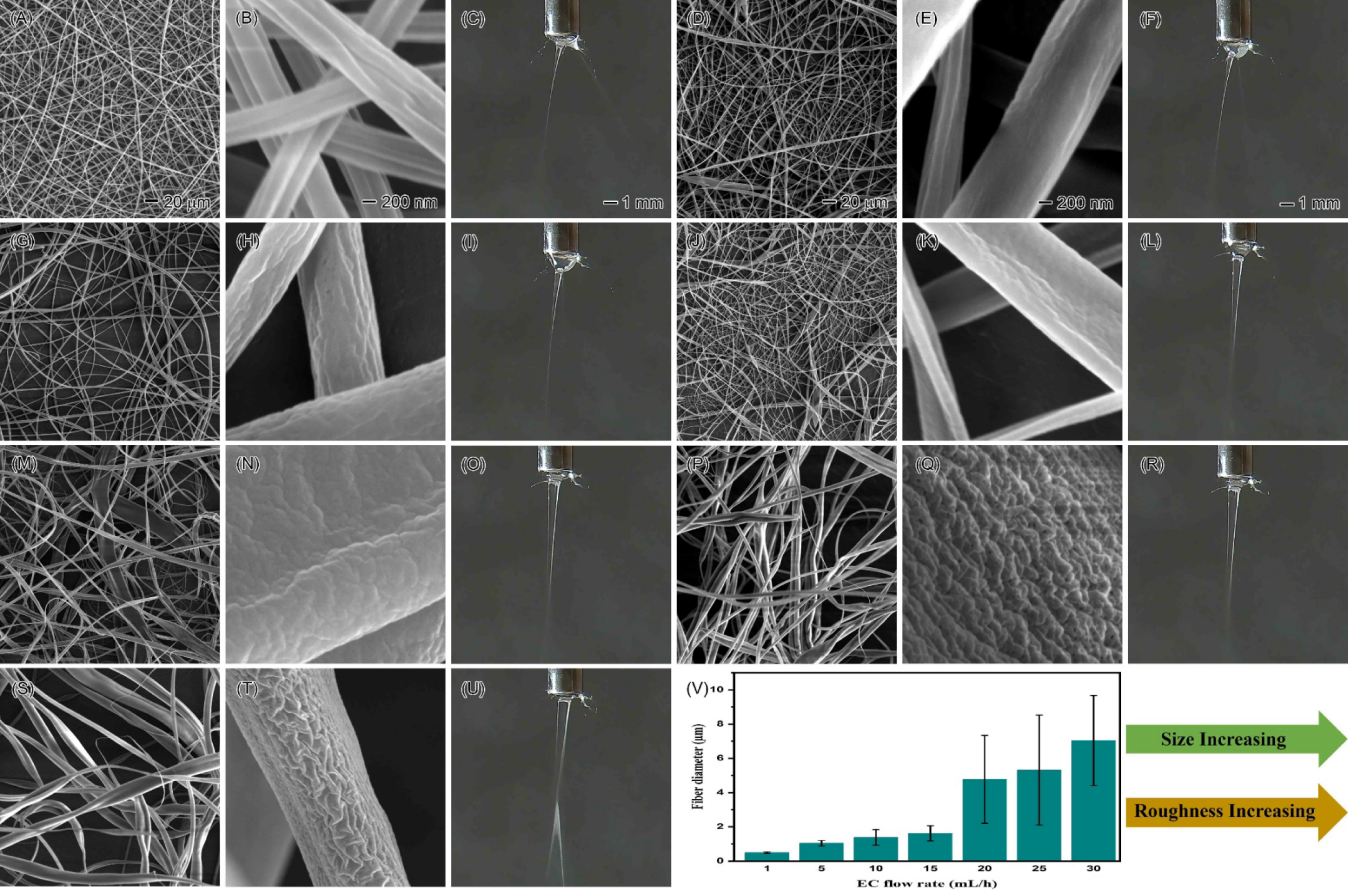
SEM images and photographs illustrating the effects of increasing the flow rate of the core EC solution with chloroform as the sheath liquid: (A–C) 1, (D–F) 5, (G–I) 10, (J–L) 15, (M–O) 20, (P–R) 25, and (S–U) 30 mL/h. (V) Fiber sizes at different core EC flow rates with a constant sheath chloroform flow rate of 0.5 mL/h. The 20 μm scale bar in panels A and D, the 200 nm scale bar in panels B and E, and the 1 mm scale bar in panels C and F apply to the corresponding images in the same column.

The high volatility of EC solvents, primarily due to the large proportion of ethanol in the 8:2 w:w mixture solvent, caused frequent clogging and disrupted the continuity of the electrospinning process.^31^ This issue significantly impeded the continuous production of the EC nanofibers. Figure 5 illustrates the effects of various EC flow rates on EC nanofibers when chloroform was employed as the sheath liquid at a constant flow rate of 0.5 mL/h. At relatively low EC flow rates (1–15 mL/h), uniform EC nanofibers with smaller sizes were formed (the mean diameters corresponding to EC flow rates of 1, 5, 10, and 15 mL/h are 0.497 ± 0.047, 1.042 ± 0.1651, 1.376 ± 0.456, and 1.617 ± 0.445 μm, respectively), and the electrospinning process remained stable and continuous without any clogging (Figure 5A–L). As the EC flow rate increased, the electrospinning process continued to be stable, although the nanofiber size became larger (the mean diameters corresponding to EC flow rates of 20, 25, and 30 mL/h are 4.773 ± 2.567, 5.317 ± 3.210, and 7.041 ± 2.630 μm, respectively) and less uniform (Figure 5M–U). The lower flow rates allowed sufficient time for the EC solvent to evaporate as the liquid jet traveled toward the collector, resulting in the formation of uniform nanofibers.^51^ Additionally, it was observed that the surface roughness of the EC nanofibers increased with higher flow rates (Figure 5V). Notably, a porous structure appeared on the surface of EC nanofibers when the EC flow rate reached 25 mL/h. This phenomenon can be attributed to several mechanisms related to the dynamics of the polymer solution and the electrospinning process. First, rapid solidification of EC fibers occurs as the flow rate increases. At higher flow rates, the EC fibers may solidify too quickly at the surface if the solvent evaporates from the outer layers while the core remains liquid. This results in internal stresses within the EC nanofibers, which contribute to surface roughness and can lead to pore formation. Second, an accelerated phase change process occurs at higher flow rates. The EC polymer solution is ejected from the spinneret more rapidly, which speeds up solvent separation from the EC polymers and contributes to surface roughness or pore formation.

When the EC solution flow rate increased from 1 to 10 mL/h, the base of the Taylor cone initially contracted toward the needle, while the Taylor cone tip (where the liquid jet ejects) significantly enlarged. This allowed more EC solution to be ejected at high speed without dripping (Figure 5C,F,I). With further increases in the EC solution flow rate from 15 to 30 mL/h, the base of the Taylor cone contracted further toward the needle and eventually flattened (Figure 5L,O,R,U), leading to a significantly larger Taylor cone tip. This increased EC nanofiber production exponentially compared to conventional electrospinning practices (0.1–1 mL/h). Several interrelated factors could have contributed to this phenomenon. First, the surface tension of the EC solution increased with higher flow rates, promoting a more stable conical spray structure and typically resulting in a smaller Taylor cone base. Second, the sheath liquid chloroform enhanced the ejection of the liquid jet by lubricating the interface between the EC solution and the sheath layer, making higher flow rates sustainable without dripping and forming a super Taylor cone tip. Other factors, such as modified electrohydrodynamic effects, might have collectively influenced the behavior of the Taylor cone in electrospinning processes.^52^

**Figure 6:**
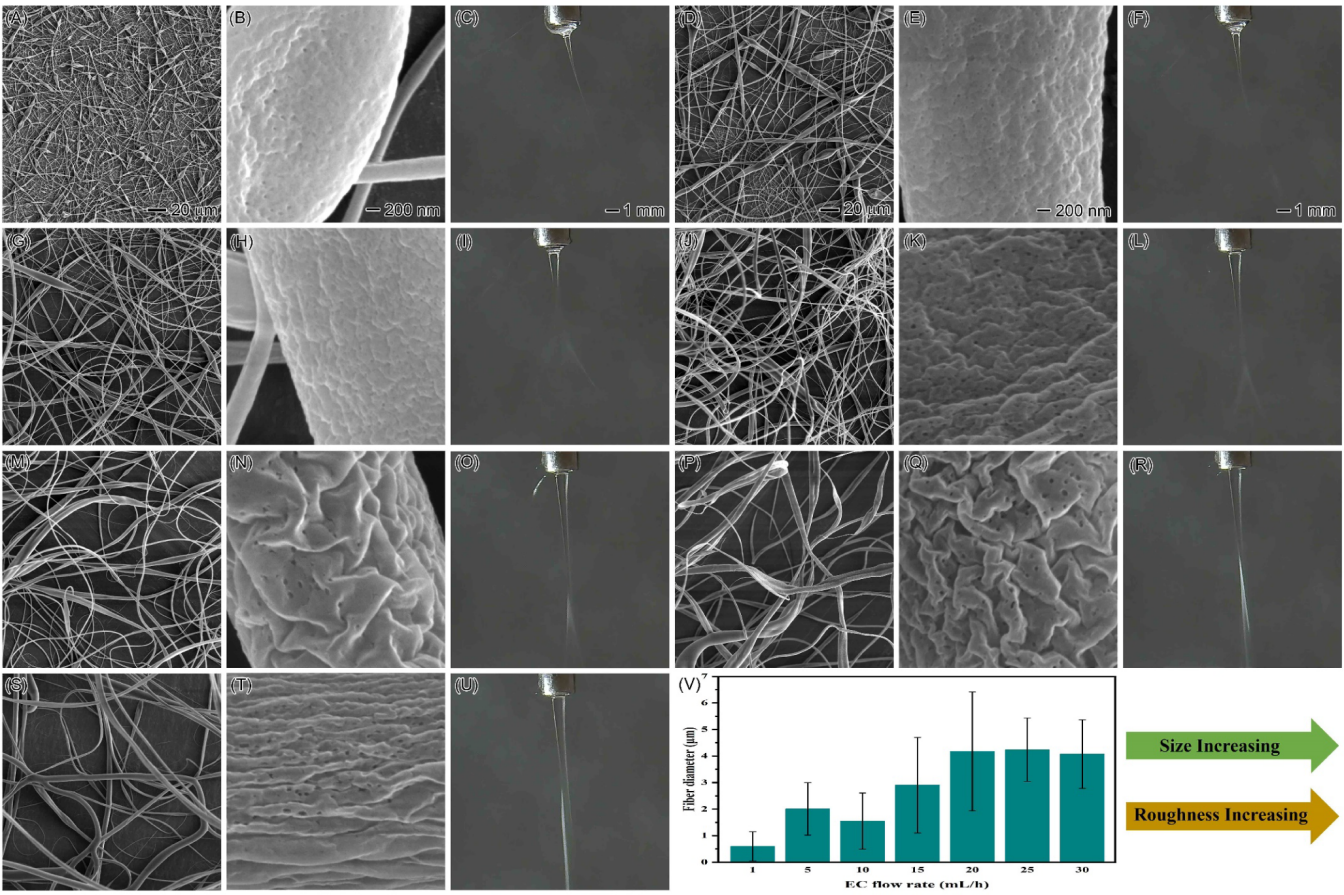
SEM images and photographs illustrating the effect of increasing the EC flow rate using ethanol as the sheath liquid: (A–C) 1, (D–F) 5, (G–I) 10, (J–L) 15, (M–O) 20, (p–R) 25, and (S–U) 30 mL/h. (V) Fiber sizes at different EC flow rates with ethanol as the sheath liquid at a constant flow rate of 0.5 mL/h. The 20 μm scale bar in panels A and D, the 200 nm scale bar in panels B and E, and the 1 mm scale bar in panels C and F apply to the corresponding images below them in the same columns.

In contrast to using potentially harmful solvents (e.g., chloroform, dichloromethane, and ether), environmentally friendly sheath liquids are preferable for increasing the flow rate of the EC solution to achieve high-speed electrospinning. Therefore, ethanol was employed as an ecologically friendly sheath liquid to improve the flow rate of the EC solution. Figure 6 illustrates the impact of different flow rates of the EC solution on both the morphology and the electrospinning process of EC fibers when ethanol was used as a sheath liquid at a constant flow rate of 0.5 mL/h. The results showed that the electrospinning process of the EC solution remained stable at low flow rates. However, many beaded nanofibers were produced (Figure 6A–F). This phenomenon is attributed to the high polarity of ethanol, which quickly diffused into the EC solution, reducing its concentration and causing the formation of beaded nanofibers due to the weakened entanglement among EC molecules. As the flow rate of the EC solution increased, the beaded structures were gradually eliminated (Figure 6G–U). This is mainly attributed to the availability of the EC polymer volume. At lower flow rates, the polymer solution may not supply enough material for continuous fiber formation. This leads to a finer jet with an insufficient polymer to maintain smooth fibers, resulting in beaded structures. Increasing the flow rate raises the polymer volume, forming thicker, more stable jets that help eliminate beaded structures. Additionally, higher flow rates increase the stretching force on the EC polymer, allowing the electric field to stretch the jet more effectively. This leads to continuous fiber formation instead of beads. Finally, higher flow rates improve the jet stability. Coarser jets are less prone to instabilities (e.g., Rayleigh instability) that can cause bead formation. Their greater inertia makes them more stable in the electrostatic field, facilitating the formation of smooth, continuous fibers. The diameter of the EC nanofibers primarily depends on the proper ratio of core EC solution to the sheath ethanol flow rates during electrospinning. Excessively high or low ratios destabilized the electrospinning process and impacted the EC nanofiber diameter. We identified an optimal flow rate ratio of 20:1 (EC:ethanol) that yielded EC nanofibers with a minimum diameter of 1.552 ± 1.058 μm. Similar to the results when chloroform was used as the sheath liquid, increasing the flow rate of the EC solution led to increased surface roughness and porosity of EC nanofibers (Figure 6V). The Taylor cone’s base and tip characteristics, using ethanol as the sheath liquid, were consistent with those observed when chloroform was used as the sheath liquid.

### Maximizing Productivity via High-Speed Electrospinning

**Figure 7:**
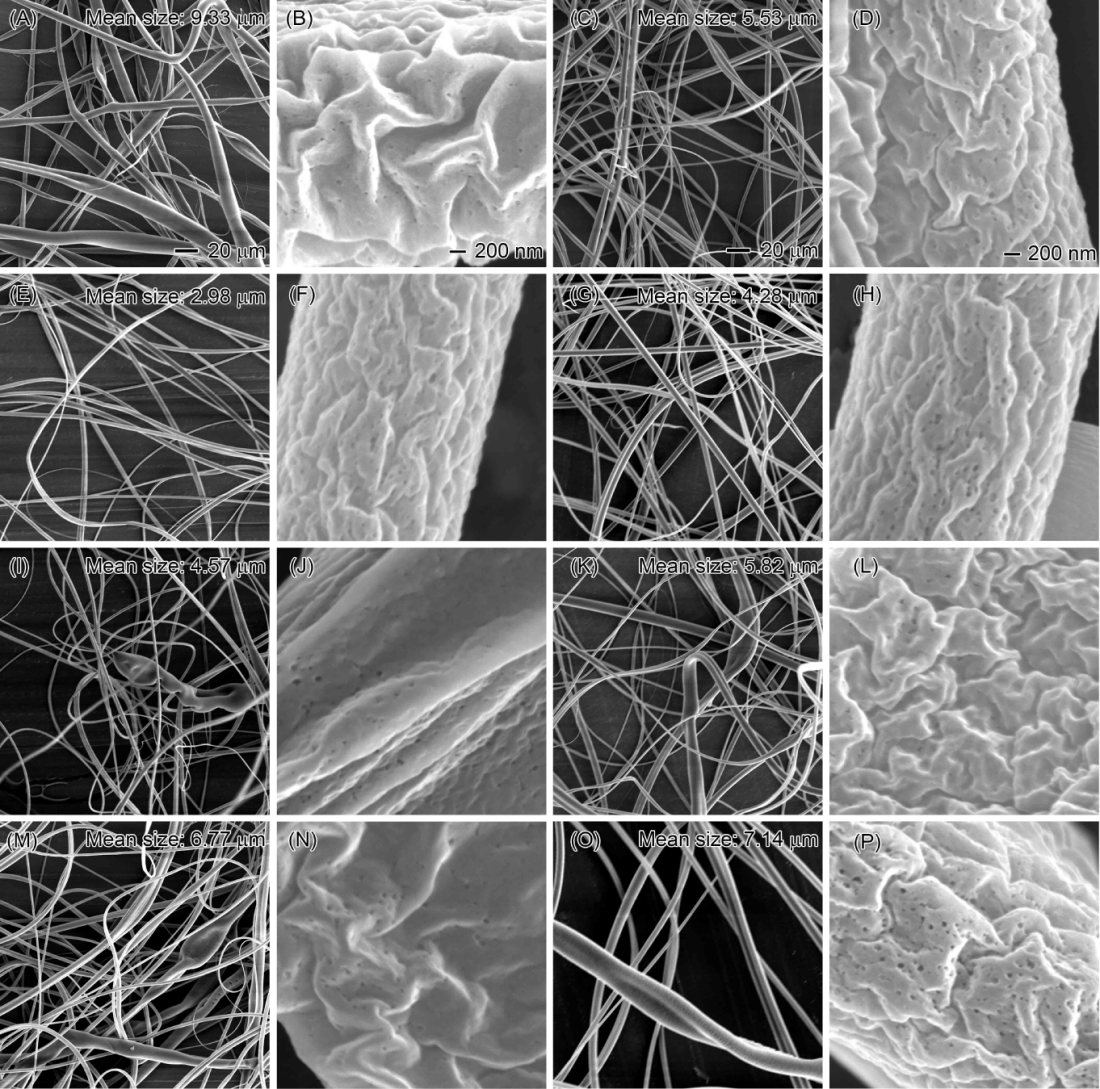
SEM images illustrating the effect of increasing sheath chloroform flow rates on the morphology of EC nanofibers. The core solution consisted of 20% EC in an 8:2 w/w ethanol/water mixture, maintained at a constant flow rate of 30 mL/h. Sheath chloroform flow rates are as follows: (A, B) 0.1, (C, D) 1, (E, F) 5, (G, H) 10, (I, J) 15, (K, L) 20, (M, N) 25, and (O, P) 30 mL/h. Scale bars: 20 μm (A, C) and 200 nm (B, D), which apply to images in the same column.

Our findings demonstrated that a small amount of sheath liquid significantly increased the level of EC nanofiber production. However, a high flow rate necessitates rapid evaporation of the solvent from the EC liquid jet before reaching the collection device; otherwise, it would facilitate the formation of beaded fibers. Employing a highly volatile sheath liquid such as chloroform can be beneficial because it accelerates solvent evaporation, leading to the formation of more uniform nanofibers. Figure 7 illustrates how increasing the chloroform flow rate affected the morphology of EC nanofibers when the EC solution was maintained at 30 mL/h. The results show that the diameter of EC nanofibers initially decreased from 9.33 μm (0.1 mL/h chloroform) to 2.98 μm (5 mL/h chloroform) and then increased to 7.14 μm as the chloroform flow rate reached 30 mL/h. During this process, the Taylor cone transitioned from stable to unstable, highlighting its critical role in controlling the high-speed electrospinning process and determining the diameter of the EC nanofibers (Figure S3).

At low chloroform flow rates (0.1–1 mL/h), inadequate wrapping of the EC solution occurred, leading to insufficient protection and resulting in the instability of the electrospinning process and coarsening of EC nanofibers (Figure 7A–D). With an increase in the chloroform flow rate, the diameter of EC nanofibers decreased significantly. Notably, at a chloroform flow rate of 5 mL/h, the average diameter of EC nanofibers was 2.98 μm (Figure 7E,F). However, further increasing the chloroform flow rate caused the EC nanofiber diameter to increase from 2.98 to 7.14 μm (Figure 7G–P). This increase is primarily attributed to the excessive presence of sheath liquid, which did not evaporate promptly and consequently encased the EC solution, leading to instability in the electrospinning process and an increase in the diameter of EC nanofibers. Thus, it is crucial to maintain an appropriate flow rate ratio between the core EC solution and sheath liquids to control the electrospinning process and the diameter of EC nanofibers. Additionally, electrospinning of EC nanofibers at high EC flow rates led to the formation of nanofibers with observable surface pores.

**Figure 8:**
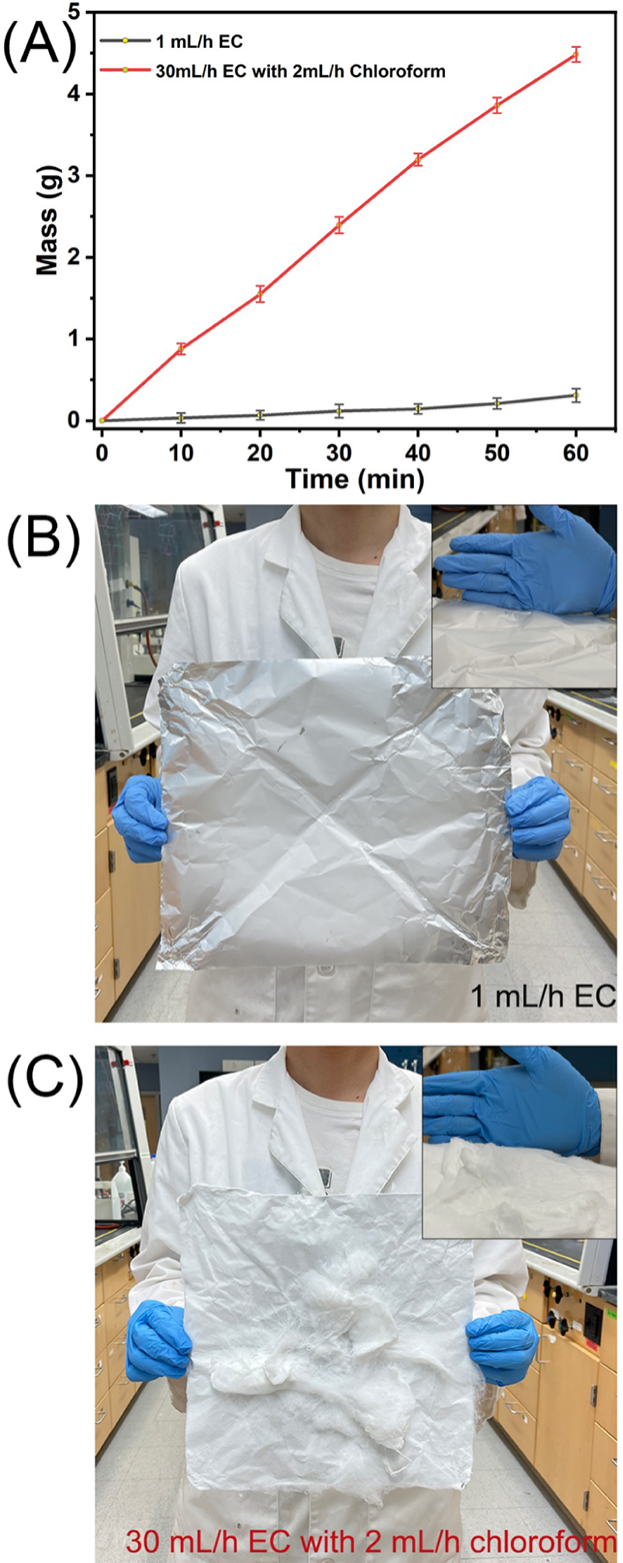
Increased yield of EC nanofibers via high-speed electrospinning compared to conventional electrospinning. (A) One-hour yield from a single spinneret for each method. (B) Photos of nanofiber membranes produced by (B) conventional electrospinning and (C) high-speed electrospinning after 1 h of operation.

High-speed electrospinning significantly increased the yield of EC nanofibers compared with conventional electrospinning. As shown in Figure 8A, high-speed electrospinning produced 4.48 g/h of dry EC nanofibers from a single spinneret, a more than 20-fold increase compared to the 0.21 g/h yield of conventional electrospinning.^53^ This yield is slightly lower than the theoretical yield of 30-fold, likely due to some nanofibers being collected on the chamber walls during the rapid production process. The visual difference in nanofiber membrane accumulation between the two methods after 1 h of operation is shown in Figure 8B,C. In our high-speed electrospinning process, the sheath liquid played a dual role, acting as both a protective barrier against rapid solvent evaporation and a lubricant to reduce the surface tension of the EC solution. This dual functionality significantly enhanced flow rates and ensured stable, continuous nanofiber generation, resulting in an exponential increase in the EC nanofiber yield. In contrast, conventional electrospinning often suffers from frequent clogging due to the rapid drying of the highly volatile EC solution within the Taylor cone, combined with its high surface tension.^31,53^ These factors pose significant challenges to continuous nanofiber production, leading to low yields and compromised quality. Our high-speed electrospinning method, utilizing a sheath liquid, successfully overcomes these limitations, demonstrating a dramatic increase in EC nanofiber yield and quality. This approach offers a pivotal strategy for the efficient and high-quality production of nanofibers from renewable polymers like EC.

### Making Porous Nanofibers Through Optimizing Core and Sheath Flow Rates

**Figure 9:**
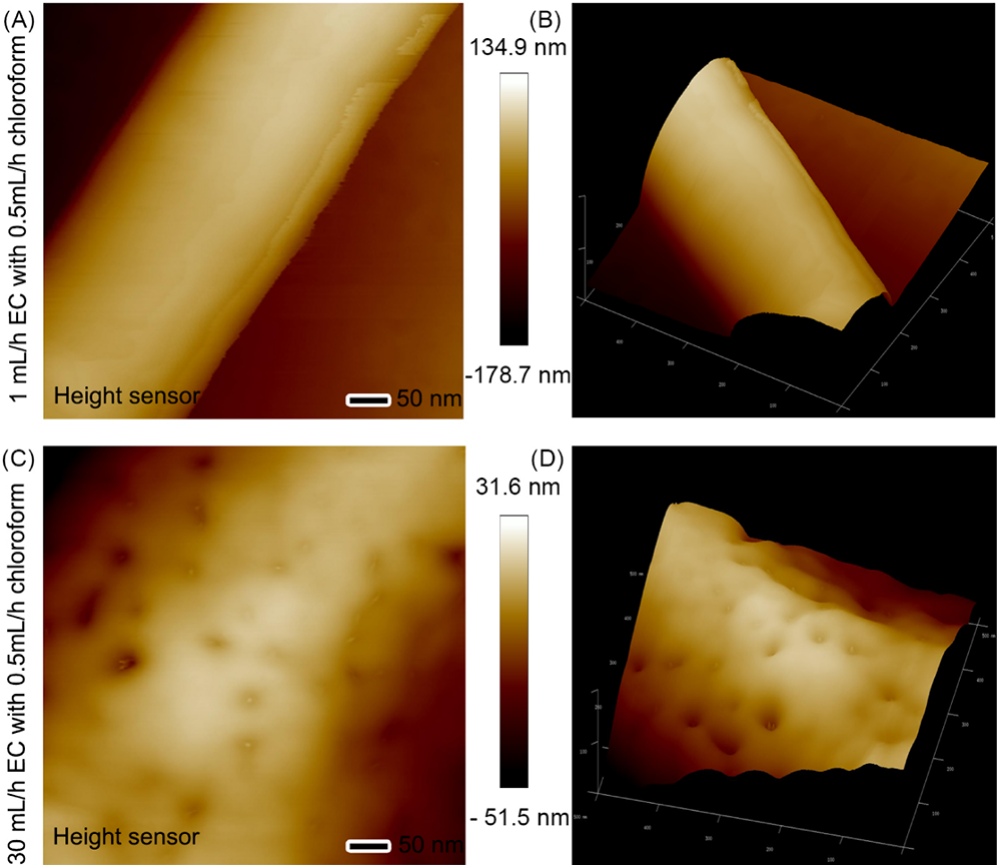
Effect of the EC flow rate on the nanofiber surface morphology. AFM height images (left) and corresponding 3D reconstructions (right) of EC nanofibers produced using a constant sheath chloroform flow rate of 0.5 mL/h and two different core EC flow rates: (A, B) 1 and (C, D) 30 mL/h.

Porous structures in nanofibers enhance performance in various applications by offering key advantages, including increased surface area, improved permeability, and enhanced functionality. The impact of the core EC flow rate on the nanofiber morphology is illustrated in Figure 9, which presents AFM height images and 3D reconstructions of EC nanofibers produced with a constant sheath chloroform flow rate (0.5 mL/h) but varying core EC flow rates. At a lower core flow rate of 1 mL/h, the resulting nanofibers exhibited a smooth surface, consistent with EC surfaces previously reported in the literature. Conversely, increasing the core EC flow rate to 30 mL/h led to the formation of nanofibers with noticeable pores or dents on their surface, likely due to the rapid evaporation of the sheath liquid and solvent.^54,55^

**Figure 10:**
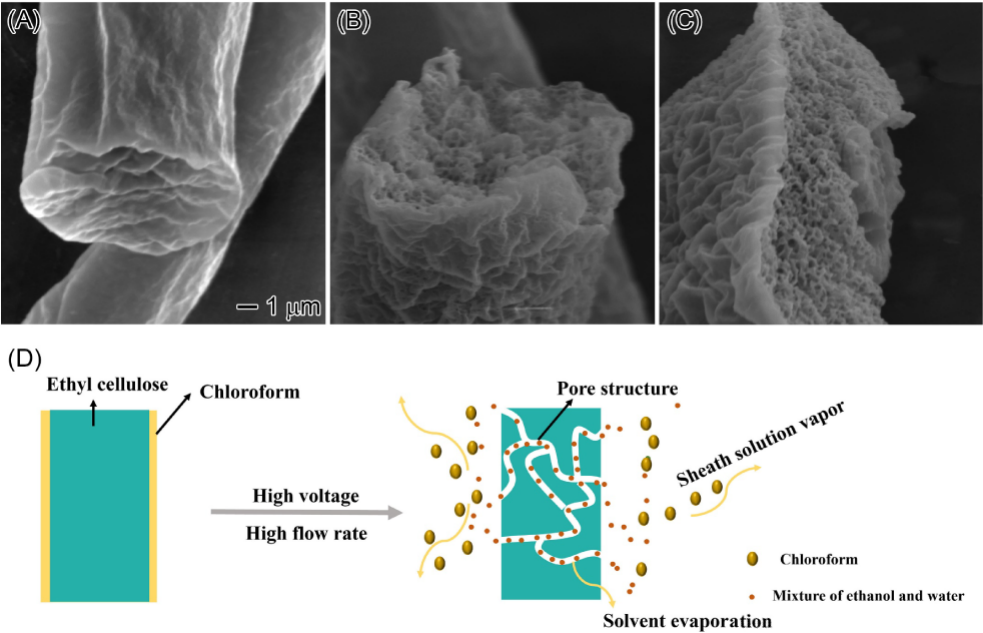
Effect of core EC flow rates on the internal structure of EC nanofibers. (A) Schematic illustration of the formation process for EC nanofibers with a porous internal structure via high-speed electrospinning. (B–D) SEM images of EC nanofibers were produced at core flow rates of 10(B), 20 (C), and 30 mL/h (D) using a constant sheath flow of 0.5 mL/h of chloroform. The core solution comprised a 20% EC solution in an ethanol and water mixture (8:2 w:w). The scale bar of 1 μm applies to all SEM images.

Our results demonstrate that increasing the core EC flow rate during electrospinning leads to the formation of pores on the surface of the resulting nanofibers. To examine the internal structure of these nanofibers, we imaged their cross sections after slicing them in liquid nitrogen. Figure 10A shows a cross-section of EC nanofibers produced with a core EC flow rate of 10 mL/h and a sheath chloroform flow rate of 0.5 mL/h. Extensive scanning of nanofiber cross sections at this flow rate revealed no internal porous structure. However, increasing the core EC flow rate to 20 mL/h while maintaining the same chloroform flow rate resulted in the development of an internal porous structure within the EC nanofibers (Figure 10B). This porosity became even more pronounced when the core EC flow rate was further increased to 30 mL/h (Figure 10C). Notably, such porous EC nanofibers, despite being highly desirable for various applications, have not been previously achieved by using conventional electrospinning techniques. Figure 10D presents a proposed mechanism for the formation of porous EC nanofibers during high-speed electrospinning. The rapid evaporation of the sheath liquid and EC solvent, driven by high core EC flow rates and high voltage, is thought to be responsible for the development of porosity within the nanofibers.^56,57^ Our findings demonstrate that increasing the EC flow rate not only enhanced the nanofiber yield but also promoted the formation of porous structures both on the surface and within the nanofibers.

**Figure 11:**
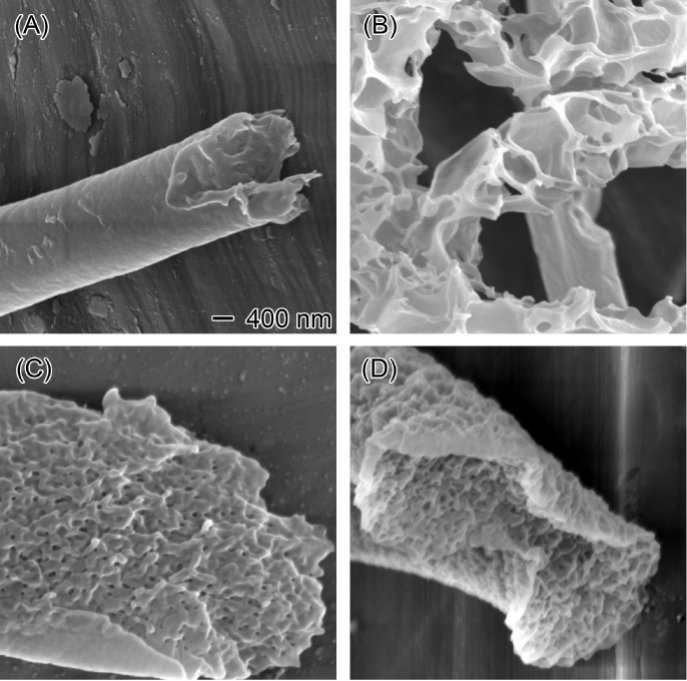
Effect of the sheath chloroform flow rate on the internal structure of EC nanofibers. SEM images of EC nanofibers (A) before and (B–D) after the removal of the surface layer. Nanofibers were produced using a 20% EC solution in an 8:2 ethanol/water mixture as the core solution (2 mL/h) and chloroform as the sheath liquid at varying flow rates: (B) 10, (C) 20, and (D) 30 mL/h. The scale bar of 400 nm applies to all images.

In addition to the core EC flow rate, the flow rate of the sheath liquid also influenced the formation of porous EC nanofibers. With a constant core EC flow rate of 2 mL/h, increasing the sheath chloroform flow rate progressively enhanced the nanofiber porosity. At 10 mL/h, the EC nanofibers exhibited a smooth, nonporous surface (Figure 11A), but upon partial removal of the surface layer with ethanol and water, a large porous internal structure was revealed (Figure 11B). At a higher sheath flow rate of 20 mL/h, dense microscale pores emerged within the lamellar structure of the EC nanofibers (Figure 11C). Further increasing the sheath flow rate to 30 mL/h resulted in a larger number of compact pores (Figure 11D). This suggests that increasing the sheath liquid flow rate accelerated solvent evaporation, promoting the formation of internally porous EC nanofibers through phase separation.^58^

### Overcoming Molecular Weight Limitations

**Figure 12:**
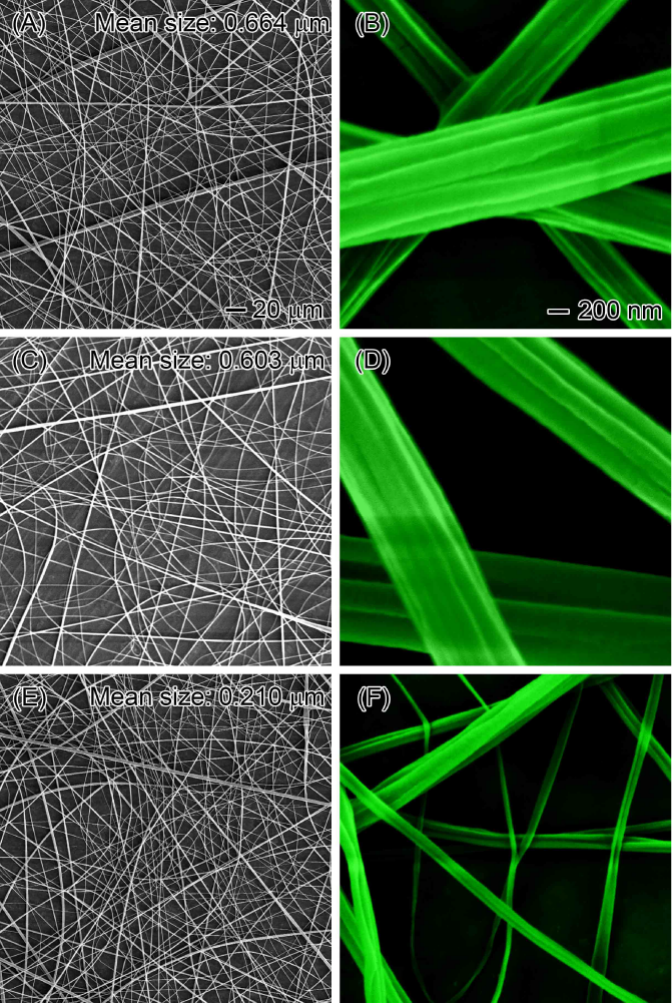
SEM images demonstrate the versatility of the sheath liquid-assisted electrospinning technique for producing EC nanofibers across a wide range of molecular weights. Molecular weight is represented by standard viscosity, with ranges of (A, B) 18–22, (C, D) 45–55, and (E, F) 90–110 mPa·s. The high-magnification images were colored to enhance the visualization of surface features and size differences.

The high-speed, sheath liquid-assisted electrospinning technique successfully produced high-yield, high-quality nanofibers from commercially available EC polymer with the lowest molecular weight (89 000 g/mol with a viscosity of 9–11 mPa·s). Higher-molecular-weight ECs present greater electrospinning challenges due to increased viscosity and surface tension. To assess the method’s broader applicability, we investigated ECs with higher molecular weights. Figure 12 presents the resulting products. Sheath liquid-assisted electrospinning (using chloroform) produced EC nanofibers with an average diameter of 0.664 μm from a 20% core solution of EC with increased molecular weight (130 000 g/mol and 18–22 mPa·s). Higher-molecular-weight ECs (224 000 g/mol or 45–55 mPa·s, and 339 000 g/mol or 90–110 mPa·s) yielded nanofibers with similar morphology but smaller diameters (0.603 and 0.210 μm, respectively). The morphological similarity likely stems from consistent intermolecular forces between the core EC solution and sheath liquid, while the decrease in diameter with increasing molecular weight suggests increased entanglement of EC chains.^59^ This successful application across a wide range of EC molecular weights confirms the versatility of sheath liquid-assisted electrospinning. Importantly, it suggests that this technique could be extended to other polymers with diverse molecular weight distributions, overcoming the limitations of conventional electrospinning and broadening the potential applications of this approach.

**Figure 13:**
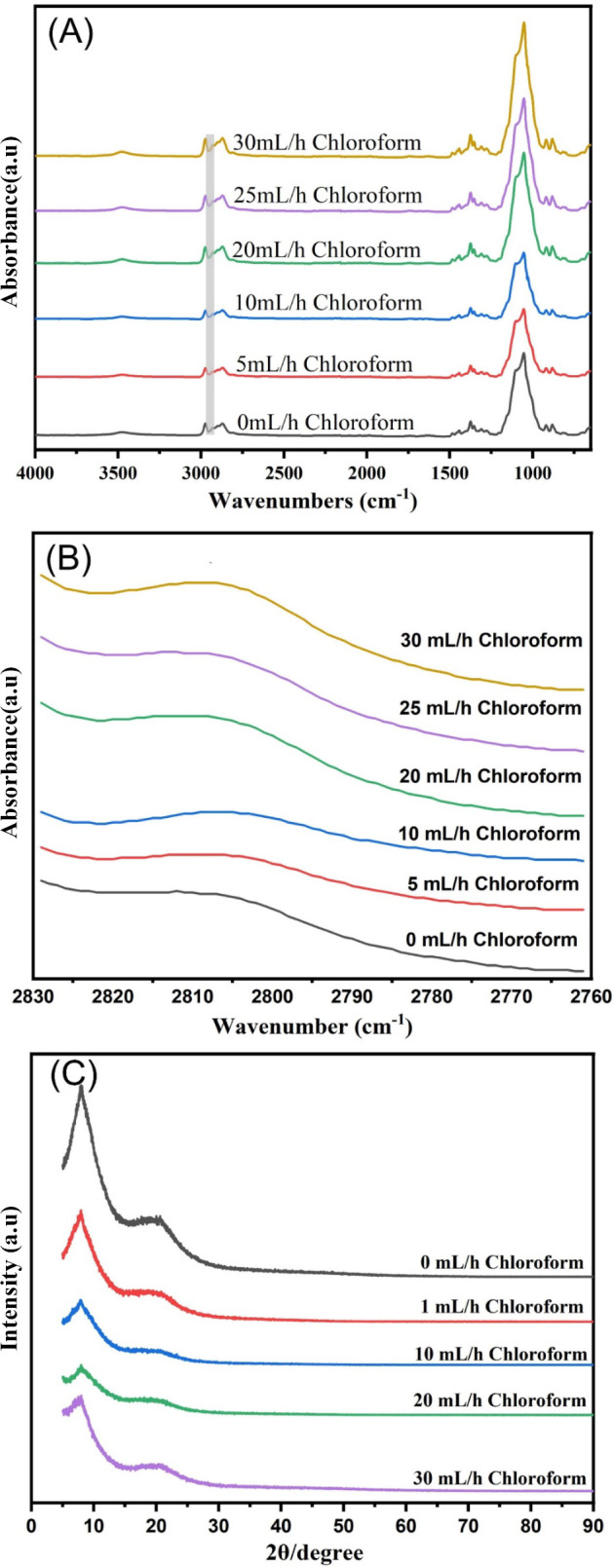
Chemical and crystalline structures of EC nanofibers produced with varying chloroform sheath flow rates. (A) FTIR spectra (overview), (B) FTIR spectra (detailed region), and (C) XRD patterns.

FTIR spectroscopy is a reliable method for analyzing the intermolecular interactions of polymers, as it identifies characteristic peak shifts and evaluates molecular compatibility. Figure 13A presents the infrared spectra of EC nanofibers prepared with varying flow rates of sheath chloroform. In the absence of sheath liquid (0 mL/h chloroform), a broad band centered at 3475 cm^–1^ is observed, corresponding to the stretching vibration of the O–H groups. Peaks at 2974 and 2869 cm^–1^ are attributed to C–H stretching vibrations. The peak at 1375 cm^–1^ is due to C–H bending, while the band at 1052 cm^–1^ corresponds to C–O–C stretching.^60,61^ The introduction of sheath liquid had a minimal impact on the overall chemical structure of the EC nanofibers, as confirmed by FTIR spectroscopy. However, a notable observation is the slight shift of the weak peak at approximately 2800 cm^–1^, corresponding to C–H stretching vibrations, with increasing chloroform flow rates (Figure 13B). This subtle shift could be attributed to trace amounts of residual chloroform retained in the nanofibers due to the high flow rates used during high-speed electrospinning. Therefore, while employing the sheath liquid-assisted technique, it is advisible to optimize the flow rate to balance the benefits of the method with the potential for solvent retention. XRD analysis was employed to investigate the impact of varying chloroform flow rates as the sheath liquid on the crystalline properties of EC nanofibers. Figure 13C presents the diffraction peaks and crystallinity of EC nanofibers subjected to different sheath liquid conditions. The EC nanofibers displayed a sharp diffraction peak at 2θ = 7.9° and a broad peak at 2θ = 20.6°.^62,63^ The diffractograms indicate that the crystalline regions within the EC nanofibers remain unchanged regardless of the sheath liquid’s flow rate. This suggests that the sheath liquid primarily influences the morphology of the nanofibers rather than their underlying crystallinity.

## Conclusions

In conclusion, this study highlights the significant advancements achieved with high-speed electrospinning, notably increasing the yield and creating novel structures of EC nanofibers using sheath liquids—achievements unattainable with conventional electrospinning techniques. To achieve high-speed electrospinning through Taylor cone optimization, we systematically evaluated a variety of sheath liquids. Our investigation into the effects of various sheath liquids revealed that their physical and chemical properties, particularly volatility, polarity, and viscosity, are crucial in determining the size, surface morphology, and internal structure of EC nanofibers. Volatility emerged as a critical factor, as insufficient evaporation of the sheath liquid caused instability in the electrospinning process, leading to nozzle clogging and dripping. Furthermore, the polarity and viscosity of the sheath liquid significantly impacted the morphology and structure of the resulting EC nanofibers. Rapid solvent evaporation at the Taylor cone tip without sheath liquids prevented the formation of a continuous liquid jet necessary for nanofiber generation, resulting in clogging and a low yield. However, our high-speed electrospinning technique, incorporating sheath liquids, maintained the integrity of the Taylor cone and sustained the rapid ejection of the liquid jet. By optimizing the Taylor cone, we significantly increased the flow rate of the EC solution from a single spinneret, boosting the nanofiber yield by orders of magnitude, which is crucial for the industrial-scale production and application of EC nanofibers. Additionally, increasing the flow rate of both EC and sheath liquids not only improved the nanofiber yield but also facilitated the development of porous structures within the nanofibers. Our technique effectively processed ECs with a wide range of molecular weights, overcoming the limitations of conventional methods. Overall, this high-speed electrospinning technique, achieved through Taylor cone optimization with sheath liquids, allows for precise control over the nanofiber size, surface morphology, and internal structure while dramatically increasing the yield. These advancements mark a significant paradigm shift in the development and industrial applications of electrospinning technology.

## Supporting Information Available

Photograph of the rapid solidification of the core EC solution within the Taylor cone (Figure S1); SEM images of EC nanoparticles (Figure S2); photographs of the effect of varying sheath chloroform flow rates on Taylor cone formation (Figure S3) (PDF)
