# Supplementary material for: High-Speed Electrospinning of Ethyl Cellulose Nanofibers via Taylor Cone Optimization
Source: ACS Appl Eng Mater. 2024 Oct 2;2(10):2454–67. doi: 10.1021/acsaenm.4c00527 (PMC11519837; doi:10.1021/acsaenm.4c00527)
Supplement: Supplementary file 1 — em4c00527_si_001.pdf [file em4c00527_si_001.pdf]

Supporting Information

For

High-Speed Electrospinning of Ethyl Cellulose Nanofibers via Taylor Cone Optimization

Qiangjun Hao,[1] John Schossig,[1] Adedayo Towolawi,[1] Kai Xu,[1] Erwan Bayiha,[1] Mayooran Mohanakanthan,[1] Derek Savastano,[1] Dhanya Jayaraman,[1] Cheng Zhang,[2] and Ping Lu[1,*]

[1] Department of Chemistry and Biochemistry, Rowan University, Glassboro, New Jersey 08028, United States

[2] Chemistry Department, Long Island University (Post), Brookville, NY 11548, United States

*Address correspondence to lup@rowan.edu (P. Lu).

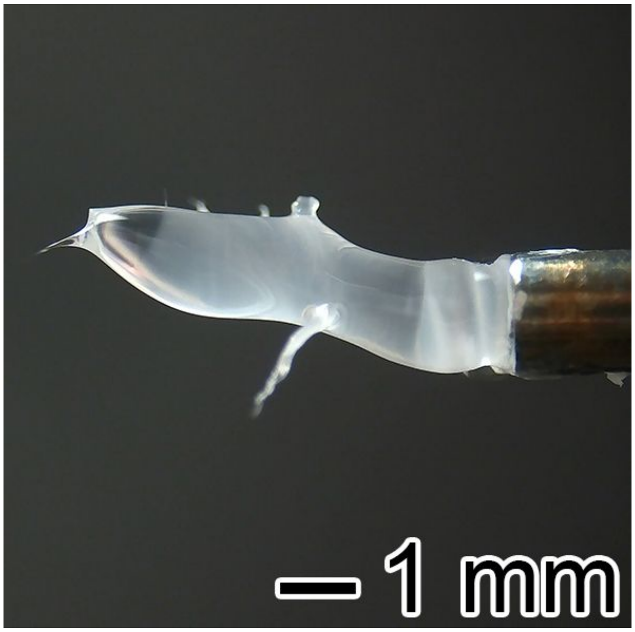

Figure S1. Rapid solidification of the core EC solution within the Taylor cone when using low-volatile water as the sheath liquid.

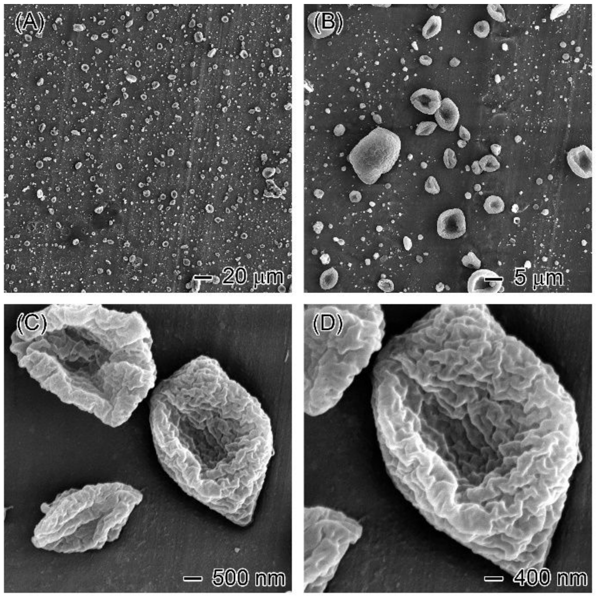

Figure S2. SEM images of EC nanoparticles produced by electrospinning a 10% EC solution (89,000 g/mol, 9-11 mPa·s).

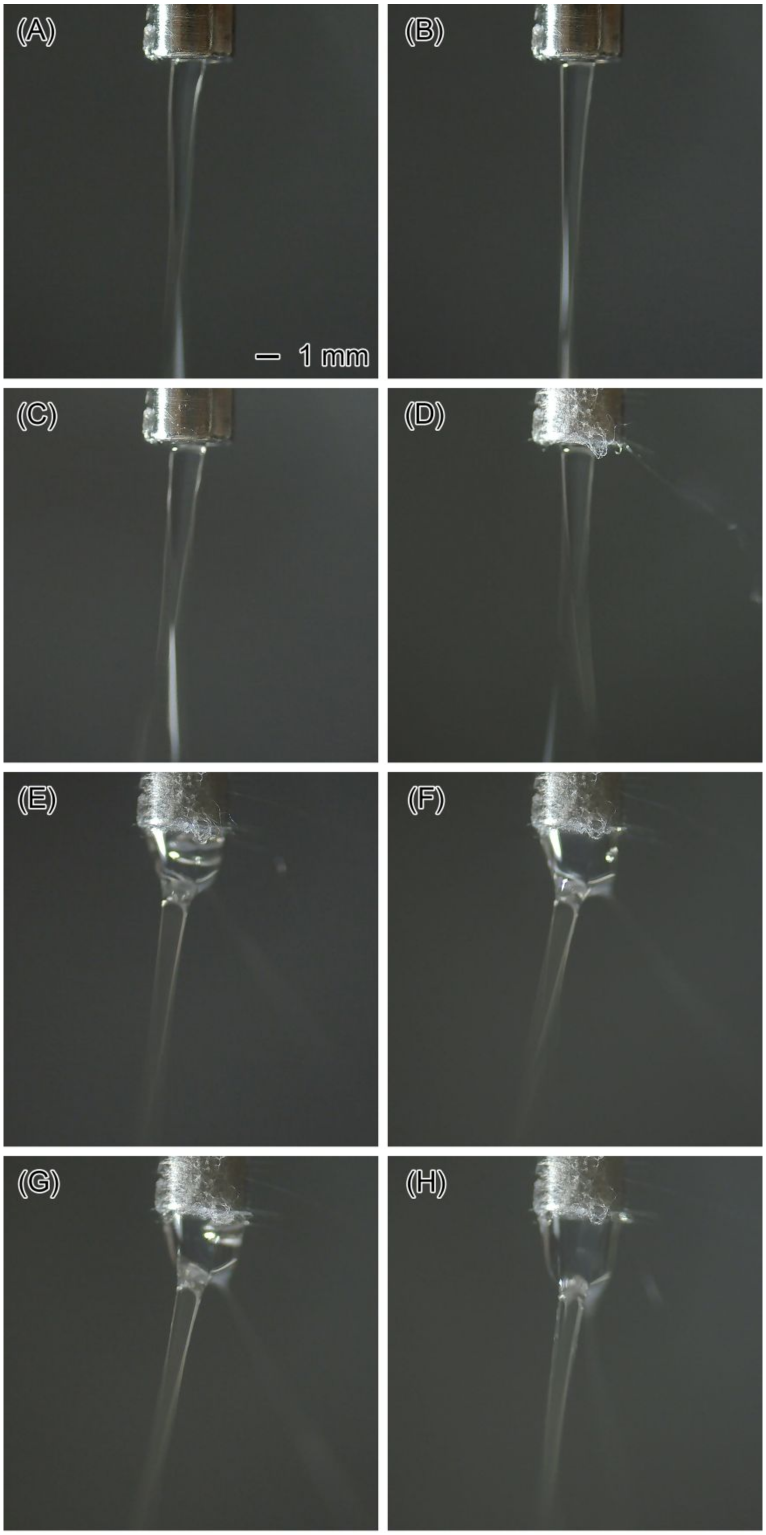

Figure S3. Photographs depicting the effect of varying sheath chloroform flow rates on the Taylor cone at a high core EC flow rate of 30 mL/h. Flow rates of sheath chloroform: (A) 0.1 mL/h, (B) 1 mL/h, (C) 5 mL/h, (D) 10 mL/h, (E) 15 mL/h, (F) 20 mL/h, (G) 25 mL/h, and (H) 30 mL/h.
